# A review on the synthesis of β-TCP using different methods for biomedical applications

**DOI:** 10.1039/d6ra02599d

**Published:** 2026-07-06

**Authors:** Taskiya Akter, Md. Habibur Rahman, Md. Shadat Hossain, Md. Mohebullah Sarker Maruf, Md. Kawcher Alam, Md. Sahadat Hossain

**Affiliations:** a Institute of Glass & Ceramic Research and Testing, Bangladesh Council of Scientific and Industrial Research (BCSIR) Dhaka 1205 Bangladesh saz8455@gmail.com; b Department of Applied Chemistry and Chemical Engineering, Noakhali Science and Technology University Noakhali Bangladesh kawcherarif00@gmail.com; c Department of Chemistry, Tulane University New Orleans Louisiana 70118 USA

## Abstract

Beta-tricalcium phosphate (β-TCP) is widely utilized in biomedical applications due to its outstanding biocompatibility and strong osteoconductive properties. Its high bioresorbability in the physiological environment allows the implanted material to undergo slow, controlled degradation over time, facilitating a gradual replacement with the body's native tissue, making it particularly suitable for bone reconstruction. A large number of studies have investigated the synthesis of β-TCP using a variety of chemical approaches, including both dry and wet approaches. Each method has distinct advantages and limitations, and these significantly influence crystallite size, crystallinity, densification behavior, shrinkage, morphology, and the overall properties of the final product. For surgical implants that require high mechanical strength, achieving high ceramic density is essential. The use of nano-sized β-TCP powders as starting materials has proven effective in producing dense ceramics. Furthermore, to preserve the resorbability of β-TCP, it is critical to obtain a pure phase. For these reasons, investigations into β-TCP synthesis have steadily increased, with a particular focus on tailoring its crystallographic properties. This review focuses on six methods for synthesizing β-TCP for bone grafting materials, dental implant applications, and bone tissue engineering. Traditional β-TCP synthesis is often criticized for generating large, non-uniform particles and secondary phases at high temperatures. In contrast, methods such as sol–gel, hydrothermal, solution combustion, and chemical precipitation are preferred for their superior control over the final product properties. We summarize the available information on each synthesis process, including benefits and drawbacks.

## Abstract 2

Beta-tricalcium phosphate (β-TCP) can be synthesized using different techniques to tune the properties for biomedical application.

## Graphical abstract

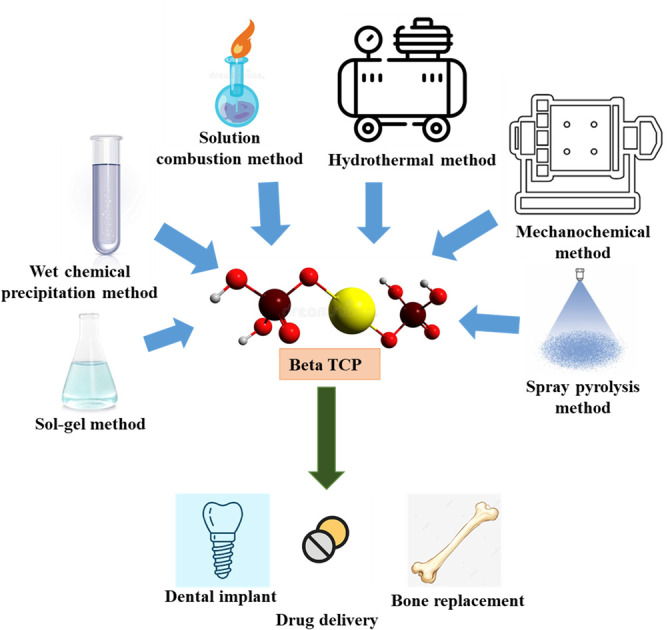

## Introduction

Significant advancements in biomaterial research have facilitated the widespread use of synthetic bone ceramic materials.^1^ Artificial materials for bone grafts, including ceramics, polymers, and metals, have emerged as viable alternatives to conventional bone replacement materials. In particular, calcium phosphate ceramics, including tricalcium phosphate, hydroxyapatite (HA), and carbonated hydroxyapatite (CHAp), are particularly well-suited for this application due to their excellent biological properties.^2–4^ Research efforts have predominantly focused on calcium phosphate ceramics, specifically tricalcium phosphate (Ca_3_(PO_4_)_2_), hydroxyapatite (Ca_10_(PO_4_)_6_(OH)_2_), and their combined forms, collectively known as calcium phosphates that are biphasic (BCP).^5–7^ In treatments that require resorbable implants, including periodontal defect management and sinus augmentation, it is vital that the material gradually degrades and is replaced by newly regenerated bone.^8,9^ Among these, tricalcium phosphate (TCP) stands out as a biodegradable and biologically active ceramic used for bone replacement. TCP exists mainly in two polymorphic phases: alpha (α) and beta (β). The β-phase is stable at temperatures less than 1125 °C, whereas the α-phase is stable between 1125 °C and 1430 °C. Owing to its high reactivity and rapid degradation in physiological environments, α-TCP is generally not preferred as a bone graft material. Consequently, the current research focuses exclusively on β-TCP.^10–12^

Regardless of composition, every biomaterial intended for medical devices must be biocompatible, defined as the material's capacity to operate in a biological system without eliciting adverse biological responses.^13–16^ β-TCP is a widely used biomaterial owing to its excellent biocompatibility as well as bioresorbability.^17^ β-TCP is considered highly effective in promoting new bone formation due to its greater solubility and faster biodegradation rate compared to hydroxyapatite (HA).^18^ The biological performance of bioceramics largely depends on their microstructural characteristics, which influence their reactivity within bone tissue. Porous β-TCP has been widely recognized for its osteoconductivity and biocompatibility. Moreover, several studies have confirmed that β-TCP exhibits a higher biodegradation rate than HA, making it a more suitable material for resorbable implants. As a result, β-TCP scaffolds are frequently utilized as bone fillers during sinus lift and socket preservation operations.^19–21^ However, for effectiveness as bone fillers, sintered bodies, or polymer composites, β-TCP must also exhibit sufficient mechanical strength. To achieve this, careful control of particle size and morphology during powder synthesis is essential. Currently, the research efforts emphasize the development of nanoscale powdered calcium phosphate with exact stoichiometry, excellent purity, and crystallinity to enhance densification, osseointegration, and bioactivity.^22^ Additionally, beyond biomedical applications, it has also gained attention for its application in thermoluminescence phosphors and high-temperature humidity sensors.^23,24^ β-TCP synthesized from calcium- and phosphate-based precursors is typically obtained by calcination at temperatures above 1100 °C.^25,26^ However, this process uses a lot of energy and makes it challenging to regulate the final product's shape.^27^ Since the introduction of secondary compromises, the inherent resorbability of β-TCP has made the use of phase-pure β-TCP crucial for biomedical applications. Pure β-TCP is a white, brittle solid, but its color may vary depending on the presence of dopants or impurities-for example, Cu-doped β-TCP appears blue to violet, Cr-doped β-TCP is green, and Mn-doped β-TCP exhibits a pink hue. The crystal structure and thermal stability are considered the most critical physical properties determining its suitability as a bone substitute.^28–30^ High-purity β-TCP is often produced by thermally converting amorphous calcium phosphate (ACP) or calcium-deficient hydroxyapatite (Ca_9_(PO_4_)_5_(HPO_4_)OH) at high temperatures.^31–33^

This review paper focuses on various wet-chemical and dry-chemical methods for synthesizing β-TCP, with particular emphasis on the solution combustion, wet-chemical precipitation, pyrolysis, mechanochemical, hydrothermal, and sol–gel methods. This study highlights that variations in source materials, synthesis methods, and reaction parameters influence the structural morphology, transforming the material from a bulk state to the nanoscale and thereby tailoring its final physicochemical properties. These methods offer distinct advantages over traditional high-temperature solid-state reactions, particularly in producing high-quality, reproducible materials with tailored characteristics. Furthermore, this paper discusses the inherent differences, complexities, and crystallographic characteristics of β-TCP obtained through each method. By adopting these synthesis strategies, researchers and manufacturers can overcome the limitations of traditional solid-state routes, such as high sintering temperatures, large particle sizes, and poor reproducibility, while producing high-quality materials with properties optimized for biomedical applications, precise control of physicochemical attributes such as crystallinity, morphology, particle size, and purity is particularly critical for the successful use of β-TCP in bone tissue engineering,^34^ bone grafts,^35^ and dental implants.^36^ Each of these methods thus provides a unique pathway to produce β-TCP with characteristics tailored to specific clinical requirements, ultimately leading to more effective and predictable bone regeneration outcomes.

### Overall mechanism

Several key properties of β-TCP must be taken into account, as well as chemical composition, structural uniformity, phase distribution, particle morphology, grain size and geometry, grain-boundary characteristics, crystallite dimensions, crystallinity level, pore structure, and surface texture.^37^ Typically, a calcium source is combined with a phosphate source, and calcium nitrate is commonly used as the Ca^2+^ source, while orthophosphoric acid or diammonium hydrogen phosphate provides the PO_4_^3−^ ions. After that, this combination is adjusted to a specific pH, often alkaline, and the solution is stirred and aged at room temperature. The precipitates are cleaned, filtered, dried, and ground into a powder after the solution has been agitated. Then the dried powder was calcined at high temperature.^38–41^1
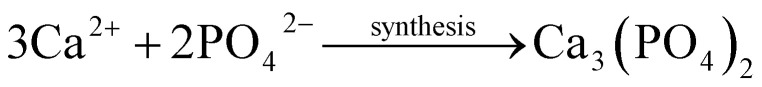


**Fig. 1:**
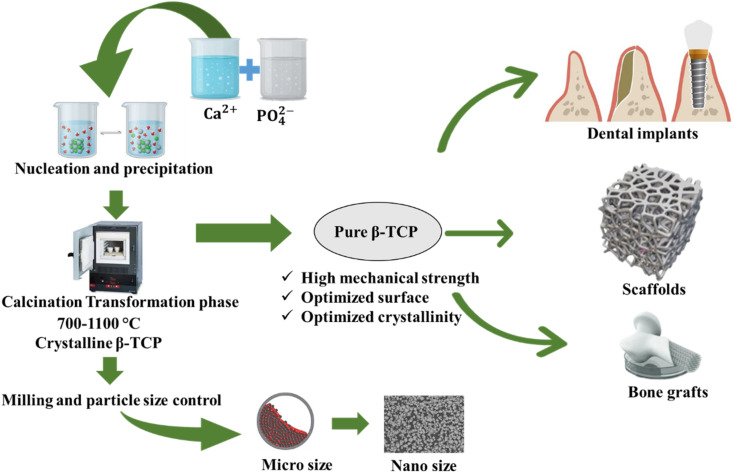
Overview of β-TCP synthesis methods and their biomedical applications.

The sol–gel method is the preferred β-TCP synthesis technique because its kinetically slow reaction rate enables the ordered arrangement of Ca and P atoms. High-quality β-TCP is achieved by controlling key factors: the firing temperature, aging period, Ca/P molar ratio, and firing time.^42,43^ Hydrothermal synthesis is a widely used, solution-reaction-based approach for preparing β-TCP nanomaterials. It concerns the synthesis of materials in an aqueous environment across a wide temperature range, from ambient to elevated temperatures.^44–46^ The wet chemical precipitation technique is a favored method for synthesizing materials, often employed for compounds such as β-TCP, because it is environmentally friendly (producing only water as a by-product), operates at low temperatures, and uses inexpensive equipment.^47^ This method's key advantage is the intimate, molecular-level mixing of reagents, which creates a highly reactive precursor powder.^48^ The full synthesis is typically a two-step process: initial precipitation followed by calcination (heating) of the precursor at temperatures exceeding 700–800 °C to induce transformation and yield a product with high phase purity. However, the resulting phase purity and the synthesis repeatability are highly sensitive to various procedural parameters.^49,50^ An independent, exothermic reaction between an oxidizer and an organic fuel in a liquid solution is used in solution combustion, a quick and energy-efficient synthesis method. The reaction's own heat production is adequate, requiring no external heat source after initiation.^51–53^ Spray pyrolysis (SP) is an efficient, liquid-phase technique for preparing highly homogeneous and stoichiometric powders. It achieves instantaneous compound formation by spraying precursor solutions into a hot furnace. SP is favored over methods such as sol–gel for its ability to produce a high, uniform yield at lower synthesis temperatures.^54–57^ Mechanochemical ball milling is a simple, cost-effective technique dating back to 1922. It uses particle collisions (deformation processes) to drive chemical reactions, synthesizing new nanosized composites or powders. It's currently recognized as a valuable alternative for preparing materials with improved biocompatibility for bone applications.^58,59^Fig. 1 provides an integrated overview of the principal synthesis routes of β-TCP and their corresponding biomedical applications. It illustrates major preparation strategies, including chemical precipitation, solid-state synthesis, sol–gel processing, mechanochemical routes, and hydrothermal methods. It highlights how these routes govern key material characteristics such as crystallinity, phase composition, particle size, and morphology, which critically influence biological responses. Building on this visual framework, the subsequent section systematically discusses the underlying synthesis mechanisms, critical processing parameters, and structure–property–application relationships of β-TCP, with particular emphasis on its relevance to bone regeneration, tissue engineering, and related biomedical applications.

### Different methods of synthesis of β-TCP

#### Sol–gel method

**Fig. 2:**
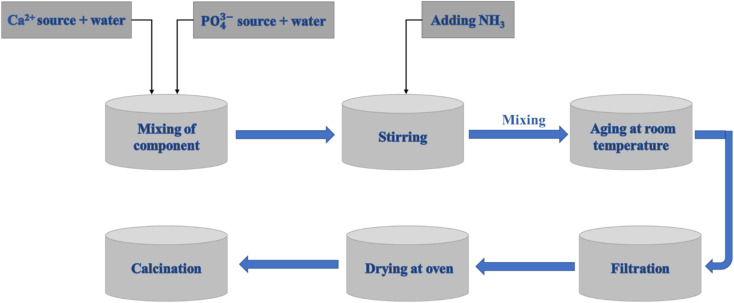
Synthesis of β-TCP using the sol–gel method.

The sol–gel method is a well-established synthesis technique for producing high-quality nanoparticles and mixed-oxide composites with excellent chemical homogeneity and controlled surface properties.^60^ It exerts a strong influence on the material's surface properties and texture. The process generally consists of five main stages: hydrolysis, polymeric condensation, aging, drying, and heat-induced decomposition.^61^ During hydrolysis, metal alkoxide precursors react in either water (H_2_O) or alcohols. When water is used as the primary reaction medium, the procedure is known as the aqueous sol–gel method, whereas the use of organic solvents under limited or controlled water conditions is known as the non-aqueous sol–gel method.^62^ The condensation stage proceeds through two principal mechanisms: Olation involves the formation of a hydroxyl (–OH–) bridge linking two cores of metal, producing metal–hydroxy–metal linkages, whereas oxolation occurs when an oxo (–O–) bridge connects two cores of metal, leading to metal–oxo–metal linkages.^63–65^ During the aging stage, the gel network gradually undergoes structural reorganization, polycondensation, and strengthening, which influence the final pore structure and mechanical stability of the material. Drying is one of the most critical stages of the sol–gel process because the removal of solvents and volatile organic species can significantly alter the gel structure through shrinkage or pore collapse. Several drying techniques, including ambient drying, freeze-drying, and supercritical drying, are employed depending on the desired material characteristics.^66^ Finally, calcination is performed to eliminate residual organic compounds, hydroxyl groups, and adsorbed water while improving crystallinity and phase formation. The calcination temperature plays a crucial role in determining the particle size, porosity, density, and overall microstructure of the synthesized material.^67^ An overall schematic representation of the sol–gel process is illustrated in Fig. 2. The sol–gel method for synthesizing β-TCP has significant advantages, including molecular-level precursor mixing, which enables high purity and homogeneity.^68,69^ Lower calcination temperatures preserve the β-TCP phase while restricting grain development. Particle size and morphology may be precisely controlled with this method, resulting in homogeneous, nanosized particles with high surface area that enhance bioactivity. It also enables simple doping with trace elements to tailor characteristics for biomedical purposes.^70,71^ This approach is straightforward, cost-effective, and environmentally benign, making it suitable for generating high-quality β-TCP for bone tissue engineering and other medicinal applications.^72^ It is also used in biomedical applications due to its ability to support bone growth, its biocompatibility, and the ease of synthesis *via* the sol–gel method.^73–75^

This method enables the synthesis of uniform nanoparticles suitable for mechanically strong and resorbable implants. For instance, in a study, β-TCP nanoparticles were synthesized by the sol–gel method, using potassium dihydrogen phosphate (KDP) as the phosphorus source and calcium nitrate tetrahydrate (CNT) as the calcium source. Approximately 0.67 M KDP and 1 M CNT solutions were prepared by dissolving in double-distilled water, and ammonia (NH_3_) was added dropwise to the mixture, yielding a white precipitate. The addition proceeded until complete precipitation was achieved at a final pH of 10. This precipitated solution was stirred, aged, filtered, and washed to remove byproducts, then dried at 40 °C and calcined at temperatures between 200 and 800 °C at a heating rate of 10 °C per minute.^76^2
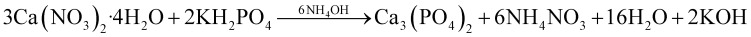


Sharp, well-defined peaks characteristic of β-TCP were observed at 800 °C, confirming the formation of the β-TCP phase. Particle size distribution analysis revealed a distorted distribution with a center of between 70 nm and 80 nm. TEM analysis showed that the β-TCP powder calcined at 800 °C exhibited a prolate spheroidal morphology. This cost-effective synthesis approach produces biocompatible nanocrystals approximately 80 nm in size, making them suitable for biomedical applications.

This method offers precise control over β-TCP size and morphology, producing smaller, more reactive particles than those in commercial powders. Using calcium nitrate, citric acid, and diammonium phosphate at pH 2, followed by calcination at 900 °C, β-TCP with a crystallite size of 115 nm and a particle size of 350 nm was obtained, compared to 150 nm crystallites and 1450 nm coarser particles in commercial samples. FE-SEM and TEM confirmed lower density and higher surface area for sol–gel β-TCP. Overall, controlling pH, temperature, and reaction time, particularly in citrate-based systems, enables the formation of homogeneous nanostructures with enhanced chemical reactivity.^77^ Also, β-TCP was synthesized by dissolving calcium nitrate tetrahydrate, citric acid (citrate : nitrate = 1 : 1), and diammonium hydrogen phosphate. The pH was maintained at 2–3 with HNO_3_ to prevent premature precipitation. The solution was stirred and heated at 70–80 °C for 3–4 h to form a transparent viscous gel, which was then calcined to obtain pure-phase β-TCP. Pure β-TCP forms at 800 °C and remains stable up to 1200 °C, with partial transformation to α-TCP at 1300 °C. At 600–700 °C, residual hydroxyapatite is present but is eliminated at higher temperatures. FESEM images show increasing agglomeration and particle growth with temperature, from uniform, nanosized particles at 800 °C to micrometer-sized particles at 1000 °C.^78^ β-TCP morphology and size are strongly influenced by the Ca/P molar ratio. Using Ca(NO_3_)_2_·4H_2_O and P_2_O_5_*via* a sol–gel route, phase-pure β-TCP was obtained at a Ca/P ratio of 1.3 after calcination at 800 °C. The crystallite size ranged from 33 to 48 nm and generally increased with the Ca/P ratio, reaching a maximum at 1.3, then temporarily decreasing at 1.4 before rising again at 1.5. Higher Ca/P ratios (up to 1.3) also resulted in larger, irregularly shaped particles, whereas impurity phases at non-optimal ratios limited crystallite growth and reduced particle size.^79^

The calcination temperature significantly affects β-TCP phase formation during the sol–gel method through a suitable precursor. In general, pure β-TCP was more stable at a calcination temperature of 1300 °C, while 600–800 °C yielded mainly β-TCP with minor α-TCP. When the calcination temperature was increased from 800 °C to 1000 °C, the particle sizes of all samples transitioned from the nanoscale to the micron range. Further morphological alterations, including the formation of α-TCP in the resultant powders, were observed at 1300 °C. DSC-TG and FTIR analyses confirmed phase purity. The microstructural analysis showed that increasing calcination temperature led to a larger particle size, especially at 800–1000 °C.^78,80^

3 Ca_9_(HPO_4_)(PO_4_)_5_(OH) → Ca_9_(PO_4_)_6_H_2_O → 3Ca_3_(PO_4_)_2_ + H_2_O

Table 1 summarizes several studies, demonstrating that the utilization of calcium and phosphorus precursors plays a critical role in the synthesis of β-TCP in either amorphous or crystalline forms. Additionally, temperature and pH significantly influence the crystal diameter and morphology.

**Table 1:** Synthesized data of β-TCP using the sol–gel method

Ca source	Ca(NO_3_)_2_·4H_2_O	Ca(NO_3_)_2_·4H_2_O	Ca(NO_3_)_2_·4H_2_O	Ca(NO_3_)_2_·4H_2_O	Ca(NO_3_)_2_·4H_2_O	Ca(NO_3_)_2_·4H_2_O	Ca(NO_3_)_2_·4H_2_O
P source	KH_2_PO_4_	(NH_4_)_2_HPO_4_	(NH_4_)_2_HPO_4_	P_2_O_5_	P_2_O_5_	(CH_3_O)_3_P	(NH_4_)_2_HPO_4_
Reaction temperature (°C)	25	80	70–80	80	37 ± 0.1	60	—
Reaction pH	10	2	2–3	—	7	—	2
Morphology	Prolate spheroidal	Agglomerated	Spherical	Porous structure	Rhombohedral	Porous agglomerated	Coral like
Amorphous/crystalline	Crystalline	Crystalline	Crystalline	Amorphous	Crystalline	Crystalline	Crystalline
Size from XRD (nm)	83 ± 6, 60 ± 4	115	—	32.97–47.82	—	—	—
Size from SEM (nm)	—	260–1000	10 000	—	—	90–150	5000
Size from TEM (nm)	70–80	4000	—	—	—	90–150	100
Calcination temperature (°C) and time	800, 600, for 30 min	900 for 3 h	1200	800 for 30 min	600–1000 for 6 h	600 for 3 h	1000 for 0.5–1 h
References	18	77	78	79	81	82	83

#### Hydrothermal synthesis

**Fig. 3:**
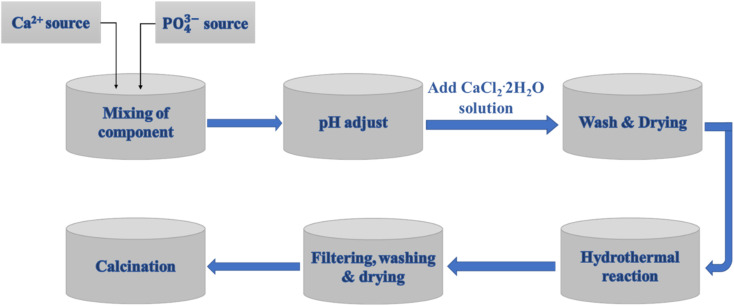
Synthesis of β-TCP using the hydrothermal method.

Hydrothermal synthesis is a solution-based method for producing crystalline materials and nanomaterials under high temperature and pressure in a sealed autoclave. This technique is especially suitable for compounds with low solubility under normal conditions.^84^ A full comprehension of hydrothermal reaction mechanisms is essential for selecting appropriate synthesis methods and for designing new materials with desired properties.^85^ These reactions typically follow a liquid-phase nucleation model, in contrast to solid-state reactions, which rely on atomic or ionic diffusion at the reactant interface.^86^ Water is frequently utilized as the solvent in the hydrothermal process,^87^ though alternative solvents may be employed to adjust crystal properties.^88^ The synthesis method relies on the significantly increased solubility and chemical reactivity of the precursors in an aqueous medium at high temperature and high vapor pressure within a sealed autoclave.^84^ As the water is heated, often reaching subcritical or supercritical states, it acts as a highly efficient solvent, dissolving otherwise insoluble solid precursors. Driven by a temperature gradient, these dissolved species are transported by convection to a nucleation site, where the solution becomes supersaturated, triggering crystal growth.^89^ This liquid-phase environment allows for precise control over the morphology and crystallinity of the final product, enabling the synthesis of high-purity nanoparticles and metastable phases at relatively low temperatures compared to traditional solid-state methods, as illustrated in Fig. 3.^90^

Process parameters such as time, pH, reactant concentration, and the presence of additives significantly influence crystal size, shape, and morphology.^91^ Surfactants also help control crystal growth and shape by sticking to specific crystal faces. They also help new crystals form, stop them from clumping together, lower surface energy, and guide crystals to assemble uniformly with precise size.^88^ Hydrothermal synthesis is especially effective for fabricating nanomaterials, including those that are thermally unstable or have high vapor pressures, as the closed system minimizes material loss.^92,93^ Furthermore, this method allows precise control over the composition and structure of nanomaterials through liquid or multiphase reactions. This special issue highlights recent advancements in the hydrothermal synthesis of nanomaterials.^94^ Hydrothermal synthesis is a flexible method for generating β-TCP in materials science and medicinal research because it provides precise control over crystal size, morphology, and phase purity, enabling synthesis in both amorphous and crystalline forms.^95,96^ The sealed reaction environment assures high purity and minimizes contamination, and water as a solvent makes the process both environmentally benign and efficient.^97^ Furthermore, this approach offers a wide range of calcium and phosphate precursors, allowing greater flexibility in materials design. Process conditions such as temperature, pH, and reaction time can influence particle size, shape, and morphology, thereby improving material performance.^98^

In a study, the pH of the precursor mixer is adjusted, and the addition of modifiers (ascorbic acid (AA)) during hydrothermal synthesis significantly alters the crystal growth of β-TCP. In this process, a precursor solution of sodium metasilicate and disodium hydrogen phosphate was treated at 150 °C and sintered at 1000 °C. SEM analysis showed that the absence of AA resulted in acicular (needle-like) crystals 5–10 µm in length, whereas the inclusion of AA, which adjusted the pH to 7.4, inhibited growth along the *c*-axis to produce spherulitic structures 5–15 µm in diameter. The resulting material featured an interconnected porous network with 5 µm pores and larger hexagonal or rectangular crystals. This demonstrates that AA serves as a critical morphological director, shifting the crystal habit from elongated needles to rounded spheres.^99^ The Ca/P atomic ratio of the precursors and temperature play a vital role in controlling the morphology and size of the final product.^100^ In one study, ACP with a Ca/P ratio of 1.25–1.55 was produced by rapidly adding aqueous ammonia to a 0.01–0.07 mol dm^−3^ calcium dihydrogen phosphate monohydrate (MCP) solution at 0 °C, resulting in gel formation. Following filtration and washing, the gel's Ca/P ratio was adjusted by varying MCP concentration and pH. In a Teflon tube, ACP was combined with 0–1.5 mol dm^−3^ solutions of carboxylic acids (formic, acetic, propionic, butyric, and valeric acids) at a 30 : 1 liquid-to-solid weight ratio. After ultrasonically treating for 5 minutes, the suspension was hydrothermally treated in an autoclave at 160–260 °C for 0–72 hours. The product was filtered and rinsed with fresh water. Formic acid excelled in the hydrothermal synthesis of β-TCP among the studied acids. The Ca/P ratio of the starting ACP significantly influenced phase formation; a lower ratio, specifically in L-ACP (1.25), suppressed hydroxyapatite (HAp) formation and favored single-phase β-TCP.^101^ Higher temperatures and longer reaction times further promoted β-TCP crystallization.^102,103^ The optimal condition for obtaining single-phase rhombohedral β-TCP was 220 °C for 3 h, and formed crystals whose size is measured at 10–30 µm. At 210 °C, a mixture of β-TCP (80 µm) and plate-like dicalcium phosphate anhydrous (DCPA) (10 µm) was observed. Above 220 °C, β-TCP reappeared in smaller sizes, accompanied by whisker-like HAp. Notably, this hydrothermal approach yielded rhombohedral β-TCP crystals, in contrast to the globular morphologies typically produced by dry synthesis methods.^104^ Another work of Othman *et al.* produced β-TCP powders from Ca(OH)_2_ and H_3_PO_4_. A 3 M solution of H_3_PO_4_ (0.3 mol) and a 0.45 M solution of Ca(OH)_2_ were mixed in a high-pressure Berghof reactor. The mixture was warmed to 70 °C and agitated at 100 rpm for 2 h. The wet precipitate was dried at 100 °C for 24 h and milled to get the as-prepared powder. The resultant powder was calcined at 900 °C for 2 h. Initially, the interaction of calcium hydroxide with phosphoric acid produced brushite (CaHPO_4_·2H_2_O), following the reaction:

4 Ca(OH)_2_ + H_3_PO_4_ → CaHPO_4_·2H_2_O

During calcination, brushite first dehydrates at around 190 °C to form monetite (CaHPO_4_), which then undergoes further transformations. The applied pressure within the hydrothermal vessel contributes to the formation of a flattened particle morphology in the synthesized powder. Hydroxyapatite and brushite were found in the as-prepared samples, according to X-ray diffraction (XRD) analysis of both as-prepared and calcined powders. Upon calcination at 900 °C, these phases transformed completely into a single phase of β-TCP. Thermal analysis showed distinct weight losses corresponding to the evaporation of adsorbed water, the dehydration of brushite to monetite, the transformation of monetite to dicalcium phosphate, and the subsequent reaction of dicalcium phosphate with hydroxyapatite, thereby forming β-TCP.^105^ These findings are supported by the chemical equations representing the sequential reactions during hydrothermal synthesis. Notably, under the initial hydrothermal conditions, the formation of brushite was favored over monetite.^106^ Another study highlights the influence of pH and temperature on β-TCP synthesis. In this study, β-TCP is synthesized by porous calcium carbonates, which are used as precursors for porous calcium phosphate, derived from the starfish species *Patiria pectinifera*.^107^ Starfish skeletons were cleaned with bleach to obtain calcium carbonate porous granules (sf-bone), which exhibited a 14 mm size and an interconnected pore structure with 10–50 nm pores. These granules were then subjected to a hydrothermal treatment using a 0.5 mol per L (NH_4_)_2_HPO_4_ solution, varying the temperature from 60–200 °C and pH (4.0 or 8.0). The conversion was highly successful at 200 °C and pH 8.0; the initial calcite phase transformed into a single-phase β-TCP after one day, while critically retaining the original interconnected porous morphology. The phase transformation was confirmed to be governed by the interplay between the rate of calcite dissolution and the rate of calcium phosphate formation, both of which are dependent on pH and temperature. This process successfully yielded β-TCP with interconnected micropores from the natural biogenic template. The presence of both micro- and macropores, due to the hollow spaces between granules, makes this material a promising candidate for use as a porous bone graft.^108^

Similarly, precursor concentration has a great influence on crystal structure. Varying concentration can change morphology as well as create variation in crystalline size.^109,110^ β-TCP was synthesized from cuttlebone using a two-step hydrothermal process. The cuttlebone was cleaned, milled, and calcined at 700 °C to obtain a calcium source, which was then reacted with phosphoric acid and varying concentrations of SDS at pH 9. Hydrothermal treatment was carried out at 60 °C for 2 h, followed by drying, calcination at 700 °C, and milling to obtain particles of ∼25 µm. FTIR analysis confirmed characteristic β-TCP phosphate bands at 567 cm^−1^ (P–O bending) and 1030 cm^−1^ (P–O stretching), with minor water and carbonate peaks and minimal influence of SDS concentration. Cytotoxicity tests using mouse fibroblast cells showed excellent cell adhesion and no cytotoxic effects, confirming the biocompatibility of the synthesized β-TCP.^111^ The studies summarized in Table 2 demonstrate the hydrothermal synthesis of β-TCP using various calcium and phosphorus precursors. The findings indicate that reaction temperature, precursor concentration, and pH significantly influence the crystal size and morphology of the synthesized β-TCP.

**Table 2:** Synthesized data of β-TCP from the hydrothermal method

Ca source	CaCO_3_	CaCO_3_	Cuttlebone	CaCl_2_	Ca(OH)_2_	α-TCP	α-TCP	α-TCP
P source	DCPA	(NH_4_)_3_PO_4_	H_3_PO_4_	MgHPO_4_·3H_2_O	H_3_PO_4_	α-TCP	α-TCP	α-TCP
Reaction temperature (°C) and time	220 for 3 h	60, 80, 100, 200 for 1 h, 1 day, 3 days, 7 days	60 for 2 h	200 for 24 h	110–130 for 1 h	40–160 for 10 h	30–240 for 20 h	180 for 9 h
Morphology	Rhombohedral	Spherical	Spherical	Block-shaped	Rod, flower-shaped	Rod shaped	Rod like	Rod shaped
Amorphous/crystalline	Crystalline	Crystalline	Crystalline	Crystalline	Crystalline	Crystalline	Crystalline	Crystalline
Size from XRD (nm)	—	—		29	—	115	—	—
SEM (nm)	10 000–30 000	1000	—	—	<5000	10 000–20 000	10 000	20 000
Calcination temperature (°C)	1100	—	700 and 4 h	1000 and 12 h	300, 500, 700, and 1 h	900 and 3 h	900 and 3 h	900 and 3 h
References	104	108	112	113	114	115	116	117

#### Chemical precipitation synthesis

**Fig. 4:**
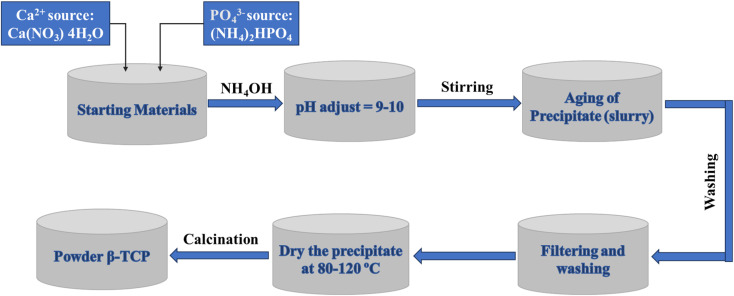
Synthesis of β-TCP using the chemical precipitation method.

β-TCP can be produced *via* multiple chemical pathways, with wet chemical precipitation being the traditional technique. Wet chemical coprecipitation is favored due to its operational simplicity, low processing temperature, and high yields of pure products at a cost-effective price.^78^ Synthesizing pure β-TCP *via* chemical precipitation requires meticulous control of multiple parameters, including reaction pH, ripening duration, temperature, and the stoichiometry of the precursor components.^118^ Even a small change in these experimental conditions can result in significant changes in the final product's composition.^119^ Most studies use calcium nitrate tetrahydrate (Ca(NO_3_)_2_·4H_2_O) and diammonium hydrogen phosphate ((NH_4_)_2_HPO_4_) as precursors.^120^ Chemical precipitation is typically a multi-step process. First, reagents containing calcium (Ca) and phosphate are combined; for example, calcium hydroxide or calcium nitrate can be used as calcium sources, while orthophosphoric acid or diammonium hydrogen phosphate can be used as phosphate sources. After that, this mixture is brought to a specific pH, often alkaline, and its temperature is maintained between room temperature and the water's boiling point.^121,122^ Subsequently, the solution is agitated to facilitate maturation, followed by washing, filtering, and drying of the precipitates, which are then pulverized into powder, as juxtaposed in Fig. 4.^123^

In a study, Massit *et al.* used several pH levels (7–9) and temperatures (30, 40, 50, 60, and 70 °C) to agitate the precipitated solution for 2 hours. The solution was then aged at room temperature for different periods (2, 24, 48, and 72 hours). The obtained XRD results of various parameters demonstrated that while the synthetic circumstances had a substantial impact on morphology, they were crucial in regulating the TCP powder's size, shape, and quality.^124^ β-TCP is typically produced through solid-state reactions or wet precipitation at temperatures of 800 °C or higher.^120^ Similarly, Kwon *et al.* calcined TCP and biphasic HA/TCP powders generated through co-precipitation. The XRD results showed that β-TCP appeared in the sample calcined at 800 °C and remained stable at higher temperatures between 800 and 1100 °C.^125^ In another work of CPS-derived β-TCP synthesis, followed by calcination at 220 °C for 120 minutes, 600 °C for 120 minutes, and 800 °C for 120 minutes. Then, the desiccated product was pulverized and sifted to acquire β-TCP powders.^126^ Ghosh and Massit *et al.* observed that the synthesized powder precipitates as CDHA, which converts into β-TCP during calcination at temperatures as high as 700 °C and above^78,124^ as described by the following equation:

5 9Ca(NO_3_)_2_ + 6(NH_4_)_2_HPO_4_ + 6NH_4_OH → Ca_9_(HPO_4_)(PO_4_)_5_OH + 18NH_4_NO_3_ +5H_2_O

6 Ca_9_(HPO_4_)(PO_4_)_5_OH → 3β-Ca_3_(PO_4_)_2_ + H_2_O

Moreover, Othman *et al.* used calcium hydroxide (Ca(OH)_2_) and phosphoric acid (H_3_PO_4_) as precursors and observed that hydroxyapatite (Ca_10_(PO_4_)_6_(OH)_2_) and monetite (CaHPO_4_) phases are formed. Upon calcination at 900 °C, the phases undergo a complete transformation into a single-phase β-TCP.^127^ Both monetite and hydroxyapatite can be made from the precursors Ca(OH)_2_ and H_3_PO_4_, according to eqn (7) and (8), respectively.

7 Ca(OH)_2_ + H_3_PO_4_ → CaHPO_4_ + 2H_2_O

8 10Ca(OH)_2_ + 6H_3_PO_4_ → Ca_10_(PO_4_)_6_(OH)_2_ + 18H_2_O

Then, when the temperature rises, monetite converts into dicalcium phosphate (eqn (9)). Subsequently, hydroxyapatite and dicalcium phosphate combine to make β-TCP (eqn (10) and (11)).^127^

9 2CaHPO_4_ → β-Ca_2_P_2_O_7_ + H_2_O

10 Ca_10_(PO_4_)_6_(OH)_2_ → 3β-Ca_3_(PO_4_)_2_ + CaO + H_2_O

11 β-Ca_2_P_2_O_7_ + CaO → β-Ca_3_(PO_4_)_2_

In another investigation, Soundharraj *et al.* confirmed the crystalline nature of the CPS-derived β-TCP particles using X-ray diffraction analysis. In that study, well-defined peaks in the diffraction pattern indicated the highly crystalline nature of the synthesized particles. From the FE-SEM micrograph, β-TCP was observed to form spherical particles with an average particle size of ∼200 nm.^128^ In another study, the average crystallite size of sintered β-TCP powder was 28.21 nm. Phosphate groups' IR peaks ranged from 900 cm^−1^ to 1160 cm^−1^. The peaks at 943.19 cm^−1^ and 972.12 cm^−1^ indicate the presence of pure β-TCP.^129^ Furthermore, Othman *et al.* employed FE-SEM to analyze the morphologies of powders calcined at five different temperatures (900–1300 °C); however, only micrographs of calcined powders at 900 °C and 1000 °C are shown.^130^ On the other hand, Lee *et al.* employed micro-CT and BET analyses of nano-sized, porous β-TCP granules, demonstrating porosity and specific surface area of approximately 75% and 2.50 m^2^ g^−1^, respectively, and an irregularly polygonal morphology.^131^ Also, β-TCP has a rhombohedral structure with the following lattice parameters: *a* = 10.429 Å, *c* = 37.380 Å.^132^ Chemical precipitation was also used to prepare β-TCP powders in methanol with aging durations ranging from 0.5 to 8 hours. The XRD patterns indicate the formation of the crystalline β-TCP (β-Ca_3_(PO_4_)_2_) phase.^133^ Besides, Puad *et al.* developed a new method that uses eggshell, converts its CaCO_3_ to CaO, and then subjects the CaO to chemical precipitation. The calcined powder was subsequently mixed with distilled water, and the pH of the resulting solution was adjusted to 8.5 with 0.6 M phosphoric acid prior to the 24 hour aging period. The precipitates were further calcined at temperatures of 300 °C, 500 °C, 700 °C, 900 °C, and 1100 °C. The XRD data indicated that 700 °C was the optimal calcination temperature for HAp, whereas samples calcined at 900 °C and 1100 °C exhibited the synthesis of biphasic HAp and β-tricalcium phosphate (β-TCP) compounds.^134^ Descamps *et al.* demonstrate in this work that the inclusion of calcium pyrophosphate or hydroxyapatite phases significantly influences the physical properties of β-TCP powders. The powders, calcined at temperatures ranging from 800 to 950 °C, were formed using slip casting, sintered at 1100 °C, and exhibited Ca/P ratios either below or above 1.5.^135^ β-TCP was synthesized *via* the CPS method at ambient temperature for 0.5 hours, during which the powders were sintered at a heating rate of 5°C min^−1^ to 800 °C. The XRD patterns demonstrate the high purity of the synthesized β-TCP. The stretching peak for PO_4_^3−^ is observed at 948–1120 cm^−1^, whereas the bending vibration peak occurs at 550–605 cm^−1^, showing that the primary component of the material is β-TCP.^136^ Alshemary *et al.* conducted a comprehensive study in which they successfully synthesized, characterized, and extracted CaP materials, such as β-TCP and hydroxyapatite (HA), for biomedical applications from diverse fish bones.^137^ In summary, β-TCP and several types of HAPs were synthesized using the wet chemical precipitation process with raw materials including calcium hydroxide, orthophosphoric acid, cow bone, and fish scale.^138^ In this method, several process parameters are involved, including the composition of the starting materials, stirring speed, stirring time, calcination temperature, and calcination soaking time. The aim of this research is to synthesize single-phase β-TCP powders by a wet precipitation process. The mechanism of phase transformation during calcination, together with the shape and thermal stability of the resultant β-TCP powder, will also be examined.^130^Table 3 summarizes various studies on the mechanochemical synthesis of β-TCP, highlighting the reaction conditions and properties of the resulting products.

**Table 3:** Synthesized data of β-TCP of Chemical precipitation synthesis

Ca source	P Sources	Reaction temperature (°C) and time	Reaction pH	Calcination temperature (°C) and time	Size (nm)	Morphology	References
Ca(NO_3_)_2_·4H_2_O	(NH_4_)_2_HPO_4_	25	>10	1000 for 2 h	—	Spherical	78
Ca(NO_3_)_2_·4H_2_O	(NH_4_)_2_HPO_4_	37 ± 0.1	7	900 for 1 h	—	Rhombohedral	81
Ca(OH)_2_	H_3_PO_4_	70 for 2 h	—	900 for 2 h	—	Agglomerated	106
Ca(OH)_2_	(NH_4_)_2_HPO_4_	30	7	900–1100	—	Spherical	118
Ca(NO_3_)_2_·4H_2_O	(NH_4_)_2_HPO_4_	25	10	775 for 5 h	59.7	Spherical	120
Ca(NO_3_)_2_·4H_2_O	(NH_4_)_2_HPO_4_	30, 40, 50, 60, and 70	7, 9	800 for 1 h	≥50–60	Spherical	124
Ca(NO_3_)_2_·4H_2_O	(NH_4_)_2_HPO_4_	—	7.4	800–1200 for 2 h	—	Spherical	125
Ca(NO_3_)_2_·4H_2_O	H_3_PO_4_	25	10	—	∼50, ∼200	Spherical	128
Ca(OH)_2_	H_3_PO_4_	70 for 2 h	—	500–1300 for 1–4 h	38.5	Aggregated	130
Ca(C_2_H_3_O_2_)_2_	H_3_PO_4_	25	—	700–800	*D*: 50	Needle-like and spherical	133
Ca(NO_3_)_2_·4H_2_O	(NH_4_)_2_HPO_4_	30 for 24 h	6.5	950	—	Agglomerated	135
Ca(NO_3_)_2_·4H_2_O	(NH_4_)_2_HPO_4_	25 for 0.5 h	—	800 for 3 h	200–300	Spherical	136
Ca(NO_3_)_2_·4H_2_O	(NH_4_)_2_HPO_4_	25	10	900 for 3 h	28.21	—	139
Ca(NO_3_)_2_·4H_2_O	(NH_4_)_2_HPO_4_	40	—	800 for 2 h	*D*: 55; *L*: 120	Rod shaped	140

#### Mechano-chemical method

The term “mechano-chemical” describes a process in which materials undergo decomposition by great mechanical force, and a new structure is formed.^141^ The mechano-chemical process can be used for material production, whereas the solid-state method yields heterogeneous particles with irregular shapes.^142^ It is a basic, eco-friendly, and economical technology and has been extensively employed in the synthesis of advanced materials.^143^ Grinding and milling are used in this approach to cause a chemical reaction through compression, shear, or friction.^144^ Two key aspects of this method stand out: (i) the products exhibit nanostructural properties, and (ii) melting is not mandatory.^145,146^ Typically, the methods employ planetary or ball mills at specific frequencies or speeds.^147^ If we consider ‘particle size and homogeneity’, this method performs best. By adjusting factors such as reagent selection, ball-to-mass ratio, powder-to-mass ratio, wet or dry milling, milling time, and rotational speed, the powder's characteristics can be improved.^148,149^

**Fig. 5:**
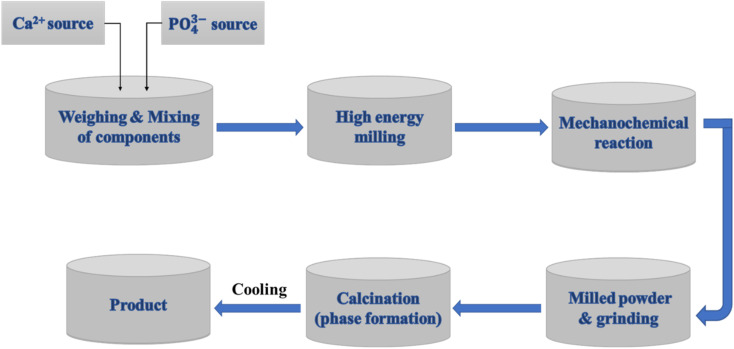
Synthesis of β-TCP using the mechanochemical method.

The mechano-chemical method uses high-energy ball milling to induce solid-state chemical reactions *via* repeated impacts and shear forces.^150^ As shown in Fig. 5, calcium (Ca) and phosphorus (P) precursors are first mixed, either in dry form or with a small amount of liquid medium, to produce a homogeneous mixture. The mixture is then subjected to high-energy ball milling, generally at rotational speeds of 350–500 rpm. The milling process reduces particle size, increases surface reactivity, and promotes atomic diffusion, thereby facilitating chemical transformation without extensive external heating. The resulting powder is subsequently calcined or sintered to enhance crystallinity, phase purity, and densification, producing stable crystalline β-TCP ceramics.^151^

In a study, Tanaka *et al.* synthesized β-TCP with CaHPO_4_/H_2_O and CaCO_3_ at a molar ratio of 2 : 1 through the mechanochemical method. The mixture was slurried in pure water and ground with zirconium particles in a pot mill for 24 h, then dried at 80 °C. Calcium-deficient hydroxyapatite was transformed to β-TCP through calcination at 750 °C for 1 hour. After sintering the β-TCP powder at 1050 °C for 1 hour, a porous block with a mean pore size of 200 µm and a porosity of 75% was formed.^152^ Heating at 700–800 °C changes Ca-deficient apatite to the low-temperature polymorph of β-TCP, resulting in water loss, as detailed by the reaction below.

12 Ca_9_(HPO_4_)(PO_4_)_5_(OH) → 3Ca_3_(PO_4_)_2_ + H_2_O

TCP can be synthesized using the solid-state reaction of calcium and phosphate precursors at 1100 °C, as shown below.^153,154^

13 CaCO_3_ + Ca_2_P_2_O_7_ → Ca_3_(PO_4_)_2_ + CO_2_

Nevertheless, sintering β-TCP ceramics to high densities is challenging because it requires elevated temperatures, and sintering above 1180 °C causes β-TCP to transform into the high-temperature polymorph (α-TCP). Many specifics remain unknown, including the exact transformation temperature, how temperature affects crystallite morphology, and the structural changes that occur throughout the transformation. The use of mechanical milling to create different calcium phosphate phases has been investigated.^155^ Therefore, depending on the milling settings, β-TCP has been synthesized *via* high-energy mechanical milling (HEMM).^156^ In a subsequent study, Tadjiev *et al.* used mechanochemical synthesis to produce biphasic calcium phosphate (BCP) powders with varying hydroxyapatite (HA) : β-TCP ratios. The starting materials were calcium hydrogen phosphate dihydrate (brushite, CaHPO_4_·2H_2_O) and calcium carbonate (calcite, CaCO_3_) at a Ca/P molar ratio of 1.67. The batch was put into a 500 mL zirconia milling jar containing 5 mm zirconia balls. A planetary mono mill was used to combine and grind simultaneously at 300 rpm for 3, 4, 5, 6, and 7 hours. XRD analysis was performed on calcined powders to identify phases and determine the HA/β TCP ratio. The combination, milled for 4 hours and calcined at 900 °C, had an 85(HA) : 15(β-TCP) ratio (BCP 85/15). The amount of β-TCP increased with milling duration.^157^ Choi *et al.* combined CaO and P_2_O_5_ precursors in a glove box with a Ca/P ratio of 1.5. A total of 10 g was then weighed into a stainless-steel vial containing seven 8 mm stainless-steel balls. The vial was sealed tightly and ground for two to sixteen hours in a high-energy mechanical mill. After milling the precursors for 8 hours, the XRD patterns indicate that β-TCP remained stable up to 900 °C in air, with a predicted crystallite size of 58 nm. Following a 10 hour heat treatment at 900 °C, the morphology of the β-TCP powders revealed a minor cluster growth to (≈2 µm) in size.^156^ In a different study, TEM and SEM analyses showed that the significant morphological changes at the higher temperature of 900 °C were attributable to both grain development and the conversion of hydroxyapatite to β-TCP. Grains with rounded corners had an average size of roughly 332 nm.^158^ Aguilar *et al.* synthesized β-TCP *via* a mechanochemical reaction using calcium carbonate and calcium dibasic phosphate as precursors. For thirty minutes, the powders were combined in a roller mill (Ca/P = 1.5). Additionally, the ball-to-powder ratio was 8 : 1, and the mechano-synthesis was run at 350 rpm for 12 and 24 hours. Ultimately, heat treatment at 900 °C for three hours produced the β-TCP phase. However, when the milling period is increased to 12 and 24 hours, the crystallite size increases to 103 and 93 nm. The XRD result demonstrated that the crystallinity of β-TCP increases with increasing milling time. The smallest particle size, 170–400 nm, was demonstrated when β-TCP was produced *via* mechano-synthesis with a milling time of 24 hours.^159^ A calcium-deficient composition with low crystallinity was produced by mechanochemical reactions using either β-TCP^160^ or possibly a combination of β-TCP and hydroxyapatite.^161^ In summary, β-TCP may be produced in high-purity crystalline phases *via* mechanochemical synthesis, owing to the chemical composition of the reagents.^162^ Additionally, longer milling times will decrease β-TCP particle size and increase crystallinity.^159^ Numerous studies on the synthesis of β-TCP *via* the mechanochemical method are summarized in Table 4, including the reaction parameters and characteristics of the resulting products.

**Table 4:** Synthesized data of β-TCP of mechanochemical synthesis

Ca source	P source	Calcination temperature (°C) and time	Size from XRD (nm)	Size from SEM	Morphology	References
CaO	P_2_O_5_	900 for 10 h	58	≈2000 nm	Cluster-like shape	156
CaO	CaHPO_4_	1100 for 2h	51.9	332 nm	Rounded corner shape	158
CaCO_3_	CaHPO_4_	900 for 3 h	93	210–1700 nm	Agglomerate	77
CaCO_3_	CaHPO_4_·2H_2_O	750 for 8 h	700	1–5 µm	Needle-like	163
CaCO_3_	CaHPO_4_·2H_2_O	1150 for 3 h	—	700 nm	Rounded	164
CaCO_3_	CaHPO_4_·2H_2_O	1200 for 1 h	5350	—	—	165
CaCO_3_	CaHPO_4_	900 for 1 h	58	2–5 µm	Agglomerate	166
CaCO_3_	CaHPO_4_·2H_2_O	750 for 8 h	700	1–10 µm	Spherulitical shapes	167

#### Solution combustion synthesis

The most popular wet-chemical method for producing oxide particles in the nanoscale and micron ranges is solution combustion synthesis (SCS), sometimes referred to as flash combustion or fire synthesis.^168,169^ Thus far, the SCS method has been utilized to generate a diverse array of solid materials. This is a novel materials-manufacturing approach characterized by a vigorous, high-temperature, self-sustained exothermic reaction, rendering the process highly energy-efficient.^170^ In 1988, Patil and Kingsley at the Indian Institute of Science in Bangalore unintentionally discovered this technique while burning a solution containing stoichiometric concentrations of urea, the high-temperature form of alumina (fuel, 8 g), and aluminum nitrate (oxidizer, 20 g).^171^ The process relies on the fire-triangle principle: combustion requires a fuel, an oxidizer, and an ignition source, and the resulting high flame temperature produces crystalline oxides. In this method, the oxidizer (such as a metal nitrate) is blended with a fuel to form an aqueous redox mixture, which is then heated until it undergoes self-sustained combustion.^172^ SCS creates a hydrogel with a homogeneous metal cation network by condensing a homogeneous aqueous mixture of precursors. During calcination, an inorganic reagent, typically a nitrate, and the fuel undergo combustion. Ammonium nitrate, sugar, citric acid (CA), tetraformyl triazine (TFTA), glycine, urea, triethylamine hydrochloride, and sorbitol are often utilized as fuels.^173,174^ The use of a fuel-oxidizer solution followed by combustion is what gives the method its name: solution combustion synthesis.^175^

**Fig. 6:**
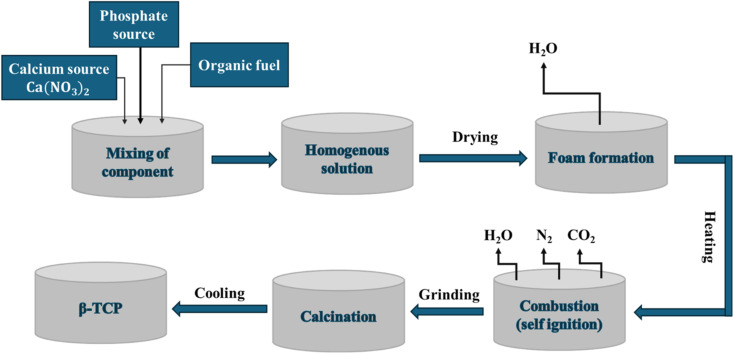
Synthesis of β-TCP using the solution combustion method.

The synthesis procedure begins with the preparation of a homogeneous aqueous solution comprising metal nitrates as oxidizers and an organic fuel, which is then heated to evaporate the solvent and form a reactive, viscous gel.^176^ Upon reaching a specific ignition temperature, a rapid, self-sustaining exothermic redox reaction between the fuel and the oxidizer produces a high-temperature flame that propagates through the precursor.^177^ Massive amounts of gaseous byproducts, including N_2_, CO_2_, and H_2_O, are released during this reaction.^178^ These byproducts expand and escape from the matrix, thereby preventing the newly formed solid from sintering.^179^ As a result, highly crystalline, fine-grained, porous nanopowders with precise stoichiometric control and a high specific surface area form almost instantly, as depicted in Fig. 6.

In a study, Bovand *et al.* synthesized β-TCP *via* a microwave-assisted solution-combustion route using 3 fuels: glycine, urea, and citric acid. Ca(NO_3_)_2_·4H_2_O served as the oxidizer, while (NH_4_)_2_HPO_4_ supplied phosphorus. The molar ratio of calcium to phosphorus was kept at 1.5, and the required fuel amounts were determined based on the following stoichiometric equation:

14 3Ca(NO_3_)_2_·4H_2_O + 2(NH_4_)_2_HPO_4_ + 2C_2_H_5_NO_2_ → Ca_3_(PO_4_)_2_ + 26H_2_O + 4CO_2_ + 6N_2_

15 3Ca(NO_3_)_2_·4H_2_O + 2(NH_4_)_2_HPO_4_ + 3CO(NH_2_)_2_ → Ca_3_(PO_4_)_2_ + 27H_2_O + 3CO_2_ + 8N_2_

16 3Ca(NO_3_)_2_·4H_2_O + 2(NH_4_)_2_HPO_4_ + C_6_H_8_O_7_ → Ca_3_(PO_4_)_2_ + 25H_2_O + 6CO_2_ + 5N_2_

The finished product was obtained by mixed combustion and calcination in a chamber furnace at 850 °C for two hours in an alumina crucible. Using citric acid as fuel made it easier for high-purity β-TCP particles to form.^180^ In another study, β-TCP was synthesized using a Ca(NO_3_)_2_·4H_2_O solution, (NH_4_)_2_HPO_4_, and urea powder. XRD results indicate that CaHPO_4_ forms at room temperature, but this phase disappears upon calcination as β-TCP develops, accompanied by a small amount of HAp up to 700 °C. A pure β-TCP phase is obtained at 800 °C and remains stable up to 1200 °C, while slight conversion to α-TCP occurs at 1300 °C. FESEM images show that particle size increases with higher calcination temperatures, shifting from the nanoscale at 800 °C to the microscale at 1000 °C.^78^ Also, β-TCP was created using a fuel mixture of succinic and citric acid. The solution pH is 1.6, indicating acidity during the process; NH_4_OH is used to raise it to pH 9.5. All precursors were calcined for 2 hours at 900 °C. Pure β-TCP is formed, according to the XRD patterns of the product made from various fuel mixture compositions. The refined lattice parameters were determined (*a* = *b* = 10.419 Å and *c* = 37.372 Å). The following is the suggested mechanism for the creation of β-TCP;^181^

17 Ca_10_(PO_4_)_6_(OH)_2_ → 2β-Ca_3_(PO_4_)_2_ + Ca_4_O(PO_4_)_2_ + H_2_O

18 Ca_10_(PO_4_)_6_(OH)_2_ → 3β-Ca_3_(PO_4_)_2_ + CaO + H_2_O

Moreover, Aghayan *et al.* explored the synthesis of biphasic HA/TCP composites *via* the solution combustion method, using urea and glycine as fuels and calcium nitrate tetrahydrate and diammonium hydrogen phosphate (Ca/P ratio of 1.5) as starting materials, with nitric acid and nitrate ions serving as oxidizers. They examined how fuel type and the fuel-to-oxidizer ratio influenced combustion behavior. When glycine was used, the combustion product consisted solely of monoclinic TCP, whereas introducing an appropriate amount of urea resulted in the formation of rhombohedral TCP.^182^ In a separate investigation, 20 mL of deionized water with Ca/P molar ratios of 1.5, 1.55, 1.6, and 1.67 was used to dissolve 0.01 mol of Ca(NO_3_)_2_·4H_2_O and varying amounts of NH_4_H_2_PO_4_. C_6_H_8_O_7_ and NH_4_NO_3_ served as fuel and oxidant, respectively. It was determined that β-TCP is one of the major phases in all samples with varying Ca/P ratios, based on the XRD patterns of the combustion products. Additionally, all combustion products were further sintered at 1150 °C for 3 hours to obtain a pure product.^183^ 90% β-TCP and 10% HAp are found in this biphasic calcium phosphate, which was synthesized using an aqueous solution combustion method. Calcium nitrate tetrahydrate and diammonium hydrogen phosphate ((NH_4_)_2_HPO_4_) were used as starting materials, with glycine as the fuel. Stock solutions of the two precursors were prepared and mixed at Ca/P ratios of 1.67 and 1.50, which are within the range suitable for producing β-TCP.^184^Table 5 summarizes the findings of several studies in which β-TCP was successfully synthesized by solution combustion synthesis. Also, the morphology and size of the synthesized β-TCP crystals are strongly influenced by reaction parameters.

**Table 5:** Synthesized data of β-TCP of solution combustion synthesis

Calcium precursor	Ca(NO_3_)_2_·4H_2_O			Ca(NO_3_)_2_·4H_2_O			Ca(NO_3_)_2_·4H_2_O	Ca(NO_3_)_2_·4H_2_O
Phosphate precursor	(NH_4_)_2_HPO_4_			(NH_4_)_2_HPO_4_			(NH_4_)_2_HPO_4_	(NH_4_)_2_HPO_4_
Ca/P	1.5			1.5			1.67	1.5
Fuel type	Glycine	Urea	Citric acid	Urea			Mixture of citric acid and succinic acid	Glycine, urea
Calcination temperature (°C) and time	850 for 2 h			800	1000 for 1 h	1300	900 for 2 h	450 for 0.5 h
Reaction pH	7.5			—			9.5	—
Crystallite size from XRD (nm)	138	69	53	—			55–65	≤50
Morphology	Coarser	Spherical	Flake-like	Equiaxed	Cylindrical	No sharp boundary	Porous agglomerated	Nodular, rod-like
References	180			78			181	182

#### Spray pyrolysis synthesis

Pyrolysis is often defined as the thermal breakdown of organic compounds in an inert atmosphere at high temperatures. The process is irreversible and causes the material to undergo pyrolysis, altering its chemical composition.^185^ Spray pyrolysis synthesis (SPS) is one of the many established pyrolysis techniques that is very useful, simple, and efficient for creating several functional nanostructures with adjustable surface chemistry and pore size. It was described as a successful method for preparing powdered materials in the 1980s.^186^ Numerous high-purity, homogenous ceramic powders with spherical morphology and extremely uniform size have been created using this technique.^187^ This procedure creates a liquid source by dissolving the inorganic precursors (beginning ingredients) in a solvent.^188,189^ The precursor solution is atomized into droplets and carried into a drying chamber, where the solvent evaporates, forming solid particles. Typically, pyrolysis is carried out at temperatures above 300 °C.^190^

**Fig. 7:**
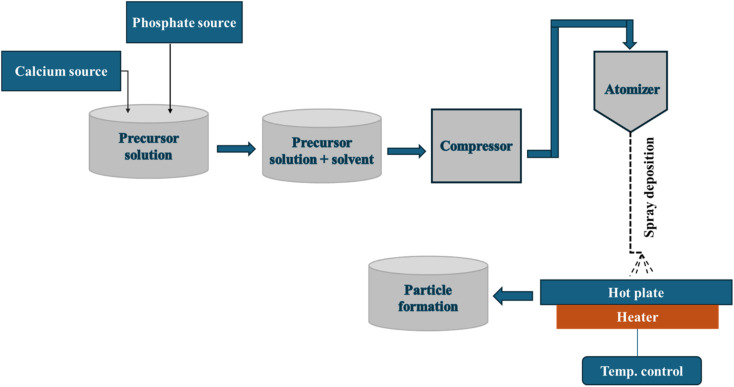
Synthesis of β-TCP using the spray pyrolysis method.

This synthesis process begins by atomizing precursor solution into a thin aerosol droplet, which is then delivered by a carrier gas toward a heated substrate, such as a hot plate.^191^ As these droplets approach the high-temperature zone, the solvent undergoes fast evaporation, resulting in a considerable contraction in droplet size and the subsequent thermal breakdown of the solute.^192^ Depending on the substrate temperature and droplet dynamics, the resulting chemical species either undergo a heterogeneous reaction directly on the surface, generating a thin solid coating, or react in the vapor phase, precipitating as fine powders, as depicted in Fig. 7.^193^ The final morphology and crystallinity of the material are ultimately dictated by the delicate balance among the solvent's boiling point, the precursors' decomposition temperature, and the ambient thermal energy of the deposition environment.

In a study, Nakanishi *et al.* synthesized β-TCP using the SPS technique. The precursor powders were prepared with a Ca/P ratio of 1.5 using calcium nitrate tetrahydrate and diammonium hydrogen phosphate serving as the sources of calcium and phosphorus, respectively. The precursor solution was prepared by dispersing 70.84 g of CNT and 26.42 g of DHP in 500 mL of deionized water. This solution was ultrasonically atomized at 1.65 MHz to form fine droplets, which were then carried through a quartz tube furnace set to 300 °C for pre-heating, 1050 °C for calcination, and 350 °C for cooling. The resulting β-TCP powders were gathered in a grounded stainless-steel tube and dried for 24 hours.^194^ Undoped, Ag-doped (2.87 and 5.75 mol%), and Ag/Zn co-doped β-TCP were synthesized *via* spray pyrolysis. The base solution used calcium nitrate tetrahydrate and diammonium hydrogen phosphate in deionized water (Ca : P ratio of 1.5), with silver and zinc nitrates added as dopants where required. All precursors were adjusted to pH 3.0 with nitric acid and stirred for 12 hours to ensure homogeneity. An ultrasonic nebulizer operating at 1.65 MHz was used to generate fine droplets of the precursor solution. These droplets were then placed in a tube furnace with zones for preheating, calcination, and cooling, set to 300, 1050, and 350 °C, respectively. Finally, the specimens were collected and dried at room temperature in an earthed stainless tube.^195^ Unlike conventional spray pyrolysis, high-temperature flame spray pyrolysis yielded spherical, non-aggregated nano-sized TCP powders. The ceramic particles at the nanoscale are created by melting and evaporating particles in a high-temperature diffusion flame. An α phase was present in the TCP powders produced immediately by flame spray pyrolysis. The fine β-TCP powders were produced by post-treating α-TCP. The XRD analysis showed that α-TCP powders remained stable at post-treatment temperatures below 700 °C. In contrast, β-TCP powders treated at 900 °C exhibited larger average crystallite sizes than those treated at 800 °C, and both treatments resulted in a pure β-TCP phase.^196^ Nanometer-sized biphasic calcium phosphate (BCP) powders with varying Ca/P ratios, achieving the desired HA/β-TCP phase balance, were produced using high-temperature flame spray pyrolysis. Ca(NO_3_)_2_·4H_2_O and (NH_4_)_2_HPO_4_ were used as precursors, with their molar ratios adjusted from 1.500 to 1.723 to achieve the desired Ca/P. XRD showed β-TCP at ratios of 1.500 and 1.543, while ratios above 1.585 produced pure HAp. Flame spray pyrolysis was not used to directly prepare phase-pure β-TCP particles. The powders produced by flame spray pyrolysis were post-treated in air at 800 °C for 2 hours. Then, monolithic TCP powder without additional phases was produced at a Ca/P ratio of 1.5. However, as the Ca/P ratio increased, the β-TCP phase fraction decreased.^197^ By using pyrolytic synthesis, Minamisawa *et al.* prepared biomimetic calcium phosphate materials using carbonized chicken manure, producing β-TCP and BCP ceramics containing both HAp and β-TCP.^198^Table 6 summarizes the findings of several studies in which β-TCP was successfully synthesized. Moreover, the morphology and size of the synthesized β-TCP crystals are strongly influenced by factors such as reaction temperature, synthesis time, precursor type, and annealing temperature.

**Table 6:** Synthesized data of β-TCP of spray pyrolysis synthesis

Ca precursor	Ca(NO_3_)_2_·4H_2_O	Ca(NO_3_)_2_·4H_2_O	Ca(NO_3_)_2_·4H_2_O	Ca(NO_3_)_2_·4H_2_O	Ca(NO_3_)_2_
P precursor	(NH_4_)_2_HPO_4_	(NH_4_)_2_HPO_4_	(NH_4_)_2_HPO_4_	(NH_4_)_2_HPO_4_	(NH_4_)_2_HPO_4_
Ca/P	3 : 2	3 : 2	3 : 2	3 : 2	3 : 2
Solvent type	Deionized water	Deionized water	Mixture of ethyl alcohol and distilled water	Mixture of ethyl alcohol and distilled water	—
Solution pH	3	3	—	7.4	—
Pre-heating temperature (°C)	300	300	—	—	300
Calcination temperature (°C) and time	1050 for 24 h	1050	600 and 900 for 3 h	800 for 2 h	1000, 1100, 1200, and 1300 for 5 h
Size from XRD (nm)	47.7 ± 1.0 nm	—	—	—	—
Amorphous/crystalline	Crystalline	Crystalline	Crystalline	Crystalline	Crystalline
Size from SEM	1.37 ± 0.57 µm	1381 ± 541 nm	57 nm	—	—
Morphology	Spherical	Spherical	Spherical	Spherical	Spherical
References	194	195	196	197	199

### Comparative overview

β-TCP can be synthesized through various methods, including wet-chemical coprecipitation, the sol–gel method, solution combustion,^197^ mechanochemical synthesis,^198^ spray pyrolysis,^199^ and hydrothermal synthesis.^200^ Among these, the sol–gel process is noted for its intrinsic advantages, such as uniform molecular mixing and the ability to produce nanocrystalline powders with a narrow particle size (approximately 70–80 nm).^201^ Using this method, pure β-TCP may be generated at a lower calcination temperature (900 °C) using the sol–gel process. However, the calcination temperature must be carefully controlled, as thermal processing strongly influences the phase purity of β-TCP. β-TCP may transform into α-TCP at high temperatures (typically above ∼1125 °C), indicating the material's temperature-dependent stability.^202,203^ However, depending on the synthesis method and processing conditions, some β-TCP powders have shown thermal stability up to 1300 °C. The high cost of the starting materials and the moisture sensitivity of some precursors limit the method's ability to maintain crystallinity during powder processing.^78,204^ β-TCP synthesized *via* this method is highly valued in biomedical engineering due to its exceptional bioresorbability, biocompatibility, and chemical purity. So, the most prominently utilized are bone graft substitutes, fillers, 3D-printed scaffolds, drug delivery systems, and dental applications.^205^ The SCS is an efficient route for single-phase HA, β-TCP, and biphasic calcium phosphate (BCP) by controlling the Ca/P ratio and annealing temperature.^206^ The choice of fuel in the SCS method is important because it affects the purity of β-TCP powders.^207^ For instance, one possible drawback of utilizing urea is that insufficient fuel oxidation may result in reddish residues in the finished product.^208^ A distinct advantage of SCS is the production of highly porous pellets (varying from 5–1000 µm pore diameter), which are advantageous for bone regeneration applications.^209^ Furthermore, this method enables the coating of calcium-phosphate films even on large-area, complex-shaped substrates.^210^ Also uses a steady flame in the burning chamber to create nanoparticles *via* either a gas-to-particle or a droplet-to-particle process.^211^ SCS is uniquely effective at producing BCP, which is used as a nanocomposite bone graft extender. Also, β-TCP nanoparticles synthesized *via* SCS are integrated into polymer cryogens to create hybrid materials for bone repair. In addition to nanoparticles' tendency to clump together, the SPS offers high crystallinity, stoichiometric control, and scalability.^212^ However, achieving a uniform particle size distribution remains a challenge. For example, using an aqueous spray solution without ethyl alcohol to prepare the product yields a bimodal size distribution spanning nanoscale and micron sizes, indicating that a uniform size distribution cannot be obtained without adjusting the solvent composition.^213^ Additionally, nanoparticles produced *via* SP exhibit a pronounced tendency to agglomerate. So, the resulting β-TCP in this method serves as the building blocks for bone scaffolds. SP is a successful “one-step” technique for applying thin, consistent calcium phosphate coatings on metallic substrates, such as titanium alloy (Ti_6_Al_4_V) dental or orthopedic implants.^214^ The hydrothermal method is an efficient process that enables morphological control using ACP precursors.^215^ A major benefit is the synthesis of porous, triphasic calcium phosphates at temperatures as low as 150 °C, resulting in highly biodegradable phases.^216^ Similarly, the microstructure and degradability of the final material can be controlled using the mechanochemical method, which provides a low-temperature way to synthesize the metastable β-TCP phase, which ordinarily requires temperatures above 600 °C.^217^ This method is cost-effective and straightforward; the addition of surfactants can further control particle shape and dispersion, preventing powder agglomeration and producing well-dispersed β-TCP powder with particle sizes between 0.1 and 0.5 µm.^218^ This method is the standard for controlling the morphology of β-TCP and can produce specific morphologies, such as needle-like crystals that closely resemble the mineral architecture of natural bone at the nanoscale. Researchers frequently use the wet chemical precipitation method due to its simplicity, precise control over process variables, and consistent production of high-purity, uniform particles.^219^ The phase regulation of β-TCP in this method is typically achieved during the washing stage with water and acetone, and a minimum calcination temperature of 775 °C is required for pure-phase formation.^220^ Recent improvements, such as the micro-dispersion process in microreactors, have eliminated classical mass transfer constraints. This upgraded approach provides a homogenous reaction environment that minimizes side reactions, yielding up to 99.8% while maintaining tight control over the Ca/P molar ratio and nanoparticle shape (80–120 nm).^221^ These highly homogeneous nanoparticles are particularly advantageous for injectable bone cements and pastes, where the consistent particle size assures predictable rheological qualities and smooth distribution through fine-gauge needles. Furthermore, the remarkable phase purity achieved through micro-dispersion makes this β-TCP an ideal choice for bioresorbable orthopedic fixation.

### Effect of different experimental parameters on the morphology and crystal structure

Key characteristics of β-TCP particles, such as crystallite size, shape, and phase composition, must be controlled for biomedical applications. Precursor reagents, impurity content, crystal size and shape, reagent concentration and mixing order, pH, and temperature are only a few of the variables that affect the bioactivity of calcium phosphate materials during synthesis.^222,223^ Particle dimensions, crystallite size, densification characteristics, shrinkage, and structural morphology are all affected differently by the different synthesis techniques.^78^ The literature offers extensive insights into the synthesis and characterization of β-TCP powder, as well as the effects of processing parameters on its properties.^224^ By fine-tuning the parameters of the precursor solution, researchers can utilize lower sintering temperatures and abbreviated processing times to influence the final product's physical state. This approach facilitates the development of a delicate, needle-like morphology characterized by high crystallinity. Ultimately, these optimized conditions are essential for maintaining the integrity of the intended nanostructure, preventing the coarse-grain growth that often occurs during more intense thermal treatments.^225^ The selection and dosage of fuel significantly dictate the phase composition and microstructure of synthesized powders. Specifically, glycine facilitates the production of α-TCP, whereas urea favors the β-TCP phase. Higher urea concentrations promote the growth of large, rod-like particles, thereby reducing the specific surface area (SSA). In contrast, increasing the HNO_3_ oxidizer content yields 500 nm agglomerates comprised of ultra-fine particles (less than 50 nm), effectively raising the SSA to 20 m^2^ g^−1^.^226^ In their research, D. Bovand *et al.* demonstrated that the choice of fuel fundamentally alters the structural dimensions of the synthesized product: citric acid yielded two-dimensional, flake-like morphologies, whereas urea yielded three-dimensional agglomerates composed of spherical primary particles. The unique flake-like porosity observed with citric acid is attributed to its role as a chelating agent. While the overall porosity of these samples stems from the significant volume of exhaust gases released during combustion, the specific geometry and scale of the pores are heavily influenced by both the fuel type and the subsequent calcination stage.^227^ In the SCS method, elevating the calcination temperature drives significant morphological evolution and phase transformation. Increasing the temperature from 800 °C to 1000 °C shifts the particle size from the nanometric range to the micrometer scale, as highly reactive spherical particles undergo neck growth, forming continuous pore channels and elongated, cylindrical grains. By 1300 °C, substantial grain growth occurs, resulting in a distinct morphology characterized by diffused, less-defined grain boundaries. This structural blurring serves as a key indicator of the phase transition from β-TCP to α-TCP.^78^ In their research on β-TCP synthesis *via* spray pyrolysis, T. Ohno and M. Aizawa demonstrated that the average powder diameter is directly proportional to the precursor solution concentration. By adjusting these ion concentrations, they successfully controlled particle diameters within a range of 0.85 to 1.4 µm. SEM analysis suggested that these larger diameters likely result from the coalescence of individual 1 µm droplets during the pyrolysis process. Additionally, the SSA decreased from approximately 11 m^2^ g^−1^ at 850 °C to 8 m^2^ g^−1^ at 1000 °C. This inverse relationship between temperature and SSA indicates that higher pyrolysis temperatures promote the accelerated growth of primary particles.^228^ Investigation into the thermal evolution of spray-pyrolyzed TCP revealed that dense, 1.3 µm spherical agglomerates undergo significant structural shifts during heat treatment. At 900 °C, the material remains purely β-TCP, though internal pore sizes begin to expand. By 1000 °C, these agglomerates develop dense outer shells, which eventually collapse at 1400 °C. This collapse, driven by the β-TCP to α-TCP phase transformation and subsequent sintering, ultimately transforms the hollow spheres into a robust three-dimensional porous network.^229^ In a study focusing on Zn-substituted TCP synthesized through spray pyrolysis, the resulting powders consistently exhibited spherical morphologies with diameters under 5 µm. The research highlighted that these particle dimensions are primarily governed by the pyrolysis temperature, carrier gas type, flow rate, and initial droplet size. Notably, the concentration of zinc in the precursor solution had no significant impact on the final particle shape or size, maintaining structural uniformity across all samples.^230^ Sol–gel synthesis of the TCP produces a diverse array of morphologies, ranging from nanoparticles and nanospheres to nanotubes and irregular agglomerates. The final architecture and dimensions are dictated by the precise calibration of wet chemical parameters, including reagent purity, pH levels, catalyst selection, and thermal processing conditions. Specifically, the introduction of organic reagents serves as a critical control mechanism, effectively templating and regulating the crystal size and geometric shape of the resulting TCP.^231^ Sanosh *et al.* observed that both crystallinity and particle size scale directly with increasing calcination temperature. TEM analysis of β-TCP powders calcined at 800 °C for 30 minutes revealed a distinct prolate spheroidal morphology. This specific geometry is attributed to anisotropic lattice changes, specifically an expansion along the *a*-axis coupled with a contraction along the *c*-axis. Such lattice distortion, particularly the *a*-axis expansion observed at lower Ca/P ratios, likely results from the increased integration of HPO_4_^2−^ ions into the β-TCP crystal structure.^232^ In a study by Fellah *et al.*, SEM analysis showed that the macropores were roughly spherical with a diameter of about 1100 µm. The microstructure varied according to the final mixture. Upon heating to 1100 °C, the β-TCP ceramic developed a microporous structure, with grains connected by concave necks, creating an interconnected network of open micropores throughout the material.^233^ Using varied conditions, particularly by changing the temperature (180 or 240 °C), affects crystallinity in the hydrothermal process; lower temperatures produce fewer distinct peaks and thus lower crystallinity.^234^ The structural and morphological properties of the β-TCP are affected by changes in temperature (110 °C and 130 °C) and duration (30 and 60 minutes) during microwave hydrothermal synthesis. In the Italiano *et al.* investigation, spherical particle agglomerates were seen for 30 minutes at a high temperature and flower-like agglomerates for 60 minutes, exhibiting distinct morphologies.^235^ In another study, it was observed that the solution's pH, the precursors' concentration, and the reaction temperature influence the composition, size, and shape of the precipitated particles.^236^ Massit *et al.* reported that raising the reaction temperature from 30 to 70 °C increased TCP crystal size (20–59 nm) and crystallinity (93–99%). pH had no effect on particle morphology. TEM showed mainly spherical, strongly agglomerated particles whose size grew slightly with longer aging. A faster Ca(NO_3_)_2_ addition rate produced smaller crystals with higher crystallinity.^237^ Another study found that β-TCP, which is made up of nanoparticles, was strongly agglomerated and nearly spherical. Particle size was found to slightly increase as ripening time increased. For (TCP-2 h) and (TCP-72 h), the observed particle sizes were around 300 nm and 400 nm, respectively. The size and crystallinity of the crystallites were shown to increase with aging time. After 24 and 48 hours, respectively, they stabilize.^238^ A study by Mirhadi *et al.* shows how pH affects β-TCP shape. This demonstrates that the β-TCP powder is highly agglomerated at pH 8, with almost spherical particles that average 150 nm in size; at pH 10.8, the powder is likewise strongly agglomerated, but its particles are smaller (80–150 nm).^239^ Calcination temperature affects grain growth, as revealed in the study by Othman *et al.*, in which the calcined powders turn into single-phase β-TCP following calcination at 900 °C, 1000 °C, 1100 °C, and 1300 °C. Nonetheless, there is a noticeable increase in grain size from around 200 nm at 900 °C to 1 µm and 2 µm at 1000 °C and 1100 °C, respectively. An apparent enhanced grain development of 8 µm is seen when the temperature is raised to 1300 °C.^240^ The chemical makeup of the raw materials and milling parameters has a major impact on the phase purity, structural features, and morphological aspects of the products in mechanochemical synthesis.^241^ During milling, the crystallite sizes progressively shrank. The crystallite diameters drop from 69 nm to 32 nm after 20 hours of milling. After sintering at 1100 °C and 20 hours of dry milling, the material displayed foamy, agglomerated particles and nanometric grains with intricate nanoscale surface features, averaging 50–100 nm in diameter. Particles with rounded edges and uneven surfaces are seen in SEM pictures.^242^

## Conclusion

β-TCP can be synthesized through various methods, each exerting a decisive influence on its phase composition, microstructure, and biological performance for biomedical applications; among them, six methods are mostly versatile: sol–gel processing, hydrothermal routes, wet chemical precipitation method, mechanochemical method, solution combustion synthesis method, and spray pyrolysis synthesis method. While all synthesis methods aim to synthesize β-TCP, the chemical pathways, thermodynamic conditions, and kinetic processes vary significantly. Variations in synthesis conditions significantly impact the phase composition, crystallinity, particle size, shape, and biological performance of the β-TCP. The sol–gel method provides superior chemical homogeneity and precise compositional control, resulting in fine-grained, highly bioactive β-TCP suitable for scaffold fabrication and bone tissue engineering. The hydrothermal method produces highly crystalline, phase-pure powders with controllable shape and size, which are necessary for improving control over *in vivo* degradation behavior. Wet chemical precipitation is the most scalable and cost-effective method; however, it requires careful control of the reaction and calcination to achieve medical-grade purity. The solution combustion method yields highly porous and reactive β-TCP that facilitates rapid cell attachment and resorption, although careful management of residual impurities is required. Mechanochemical synthesis is a solvent-free, energy-efficient method for generating bioactive β-TCP with minimal processing steps, provided that contamination from grinding material is well controlled. Alongside these conventional methods, novel approaches such as solution combustion synthesis, direct fabrication, hybrid scaffolding, spark plasma sintering, and microwave-assisted synthesis have garnered increasing attention for their potential to integrate with cutting-edge biomedical manufacturing technologies and to offer shorter processing times, greater energy efficiency, superior mechanical strength, and improved phase control. Overall, sol–gel and wet-chemical precipitation procedures are widely regarded as the most versatile and reliable strategies for β-TCP synthesis, offering the best balance of material quality, scalability, and applicability for biomedical applications. Future studies ought to focus on developing eco-friendly, biocompatible, and energy-efficient strategies, together with modern fabrication and processing approaches, to improve scalability, reduce production costs, and expand the clinical and translational potential of β-TCP.

## Author contributions

Taskiya Akter and Md. Habibur Rahman collected the data and wrote the draft and original manuscript. Md. Sahadat Hossain conceived and designed the review, analyzed the data, and assisted in writing the manuscript. Md. Kawcher Alam, Md. Mohebullah Sarker Maruf and Md. Shadat Hossain assisted in collecting data.

## Conflicts of interest

There are no conflicts to declare.

## Data Availability

Data will be made available on request.
